# Coexistence of splenic hemangioma and vascular malformation of the vertebrae

**DOI:** 10.1186/s13104-016-1860-6

**Published:** 2016-02-09

**Authors:** Hasan Jalaeikhoo, Mehdi Ariana, Seyed Mohammad Hossein Kashfi, Pedram Azimzadeh, Ahmad Narimani, Masoomeh Dadpay, Manouchehr Keyhani

**Affiliations:** AJA Cancer Research Center (ACRC), AJA University of Medical Sciences, Tehran, Iran; AJA Trauma and Surgery Research Center, AJA University of Medical Sciences, Tehran, Iran; Gastroenterology and Liver Diseases Research Center, Research Institute for Gastroenterology and Liver Diseases, Shahid Beheshti University of Medical Sciences, Tehran, Iran; Hematology and Oncology Research Center, Vali Asr Hospital, Tehran University of Medical Science, Tehran, Iran

**Keywords:** Autopsy, Cavernous hemangioma, Magnetic resonance imaging

## Abstract

**Background:**

Cavernous hemangioma is an encapsulated mass of dilated, endothelial lined vascular channels filled with slowly flowing blood. Cavernous hemangioma of the spleen is a rare condition with less than 100 reports so far. Hemangioma of the vertebral is a benign vascular legion around one or two vertebrae. These are usually asymptomatic and discovered incidentally. In this study we reported an extreme rare case of splenic hemangioma coexistence with vascular malformation of the vertebrae. To our knowledge this is the first report of coexistence of splenic hemangioma and hemangioma of the vertebra.

**Case presentation:**

A 20-year-old iranian male with splenomegaly, abdominal pain, diarrhea and pancytopenia who was first highly suspicious for malignancy referred to our center for evaluation of the diagnostic workup. After full examination we detected a very rare case with a giant, solitary cavernous hemangioma of the spleen and multiple hemangiomas in his vertebrae. Histopathology of the spleen showed a large cavernous hemangioma occupying almost the entire spleen with large areas of infarction necrosis with multiple hemangiomas of the vertebrae.

**Conclusion:**

It is extremely rare to have a splenic hemangioma concurrent with vertebra hemangioma and this is clinically very important to consider splenic hemangioma in differential diagnosis of splenomegaly for a better therapeutic management in related patients.

## Background

Hemangioma is the most common benign primary neoplasm in spleen and the prevalence ranges from 0.3 to 14 % at autopsy [1, 2]. This vascular malformation is very common in adults during third to fifth decade which grows slowly [1]. These benign lesions of spleen are usually asymptomatic and detected incidentally; however, thrombocytopenia, anemia and coagulopathy could co-existence with large lesions [3]. Cavernous hemangioma of the spleen is considered as a rare disorder with less than 100 cases reported so far [4]. Rupture, infarction, hypersplenism, malignant degeneration, thrombosis, infection with abscess formation and partial calcification of the vascular spaces are complications of the splenic hemangiomas. It has been demonstrated that 25 % of cases with large hemangiomas (larger than 4 cm), develop splenic rupture [5]. With atypical features in sonographic procedure, it makes the diagnosis very difficult [6]. MRI is considered as a great tool for diagnosis and identification of splenic diseases including splenic hemangiomas. While asymptomatic hemangiomas do not warrant any treatment, symptomatic or large lesions are treated by splenectomy. Vertebral hemangiomas (VH) are benign vascular legions around one or two vertebrae. These lesions are usually originated from dysembryogenetic or hamartomatous [7]. In autopsy the rate of vertebral hemangiomas is 10–12 %. These benign lesions account 2–3 % of all spinal tumors [8]. They usually present incidentally in the lower thoracic and upper lumbar spine and as a result those might lead to vertebral compression or vertebral collapse [9]. Vertebral hemangiomas do not unusually associated with any symptoms and only up to 1.2 % of lesions presented with obvious symptoms. Even though, the treatment of this symptomatic vertebral hemangioma has been controversial [10–12]. In this condition, the commonest symptom is back pain followed by weakness of the lower back and legs. In this study we reported an extreme rare case of splenic hemangioma coexisted with vascular malformation of the vertebrae. To our knowledge there has not been any report of simultaneous presentation of splenic hemangioma and hemangioma of the vertebra so far.

## Case presentation

This is a report of a very rare case of a giant, cavernous hemangioma of the spleen with multiple hemangiomas in the vertebrae. A 20-year-old iranian male referred to the hematology department, Imam Reza hospital, Tehran in Feb 2010 with occasional pain in the left upper abdomen which lasted for 2 months. The abdominal pain radiated to lower back. He gave the history of 13 kg weight loss during last 7 months.

There was no history of fever and urinary symptoms. Occasionally night sweating was in his past medical history. He had the same type of abdominal pain 10 years ago. There were not any important positive findings in his familial history. The general physical examination was unremarkable except for the spleen which was grossly enlarged, extending to 4 cm under umbilicus. Blood counts showed a mild pancytopenia. Serology of viral hepatitis and HIV, liver and renal function tests and hemoglobin electrophoresis did not show any abnormality. Bone marrow aspiration was normal. Ultrasonography of spleen showed multiple diffuse cystic lesions, suspicious to cavernous hemangioma or splenic abscess.

**Fig. 1:**
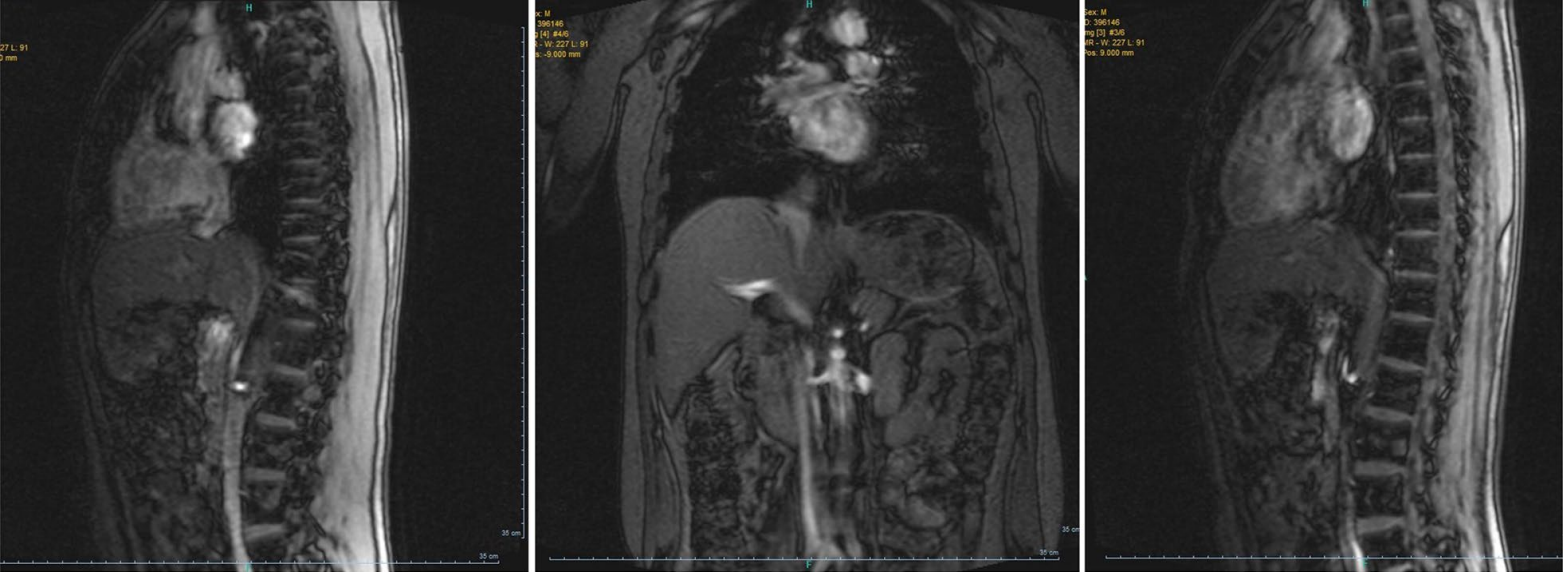
Without contrast magnetic resonance (MR) imaging of lumbosacral spine with multiple abnormal bone marrow signal

**Fig. 2:**
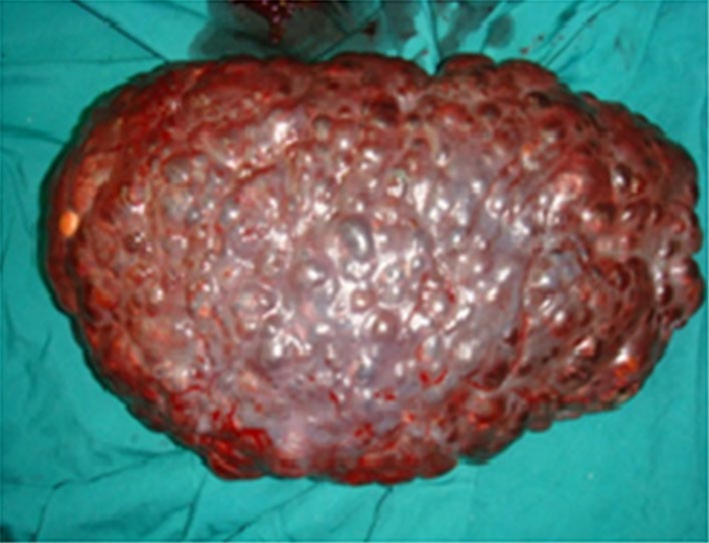
The huge cavernous hemangioma of the spleen with multiple hemangiomas in the vertebrae. The surface of the spleen was multicystic. Splenectomy was performed. The resected specimen and imprint cytology was sent for pathology evaluation

Contrast-enhanced computed tomography (CECT) of the abdomen showed a large, lobulated mass with multiple hypodense round lesions in different sizes in its counter in anatomic site of spleen, suggestive of splenic hemangioma or metastasis. There was not any free fluid in his abdomen. Without contrast magnetic resonance (MR) imaging of lumbosacral spine showed multiple abnormal bone marrow signal alteration through vertebral body in favor of metastatic lesion referred as a vertebral hemangioma (Fig. 1). Upper GI endoscopy and CT scan of thorax and brain were normal. The patient was explored by a left subcostal incision under general anesthesia. The lesions in vertebral hemangiomas at without contrast MRI and in both sequences T1, T2 has a low signal and in t1 has a high signal. There was a giant splenomegaly with a large solid lesion confined to the lower half of the spleen. Splenectomy was performed (Fig. 2).

**Fig. 3:**
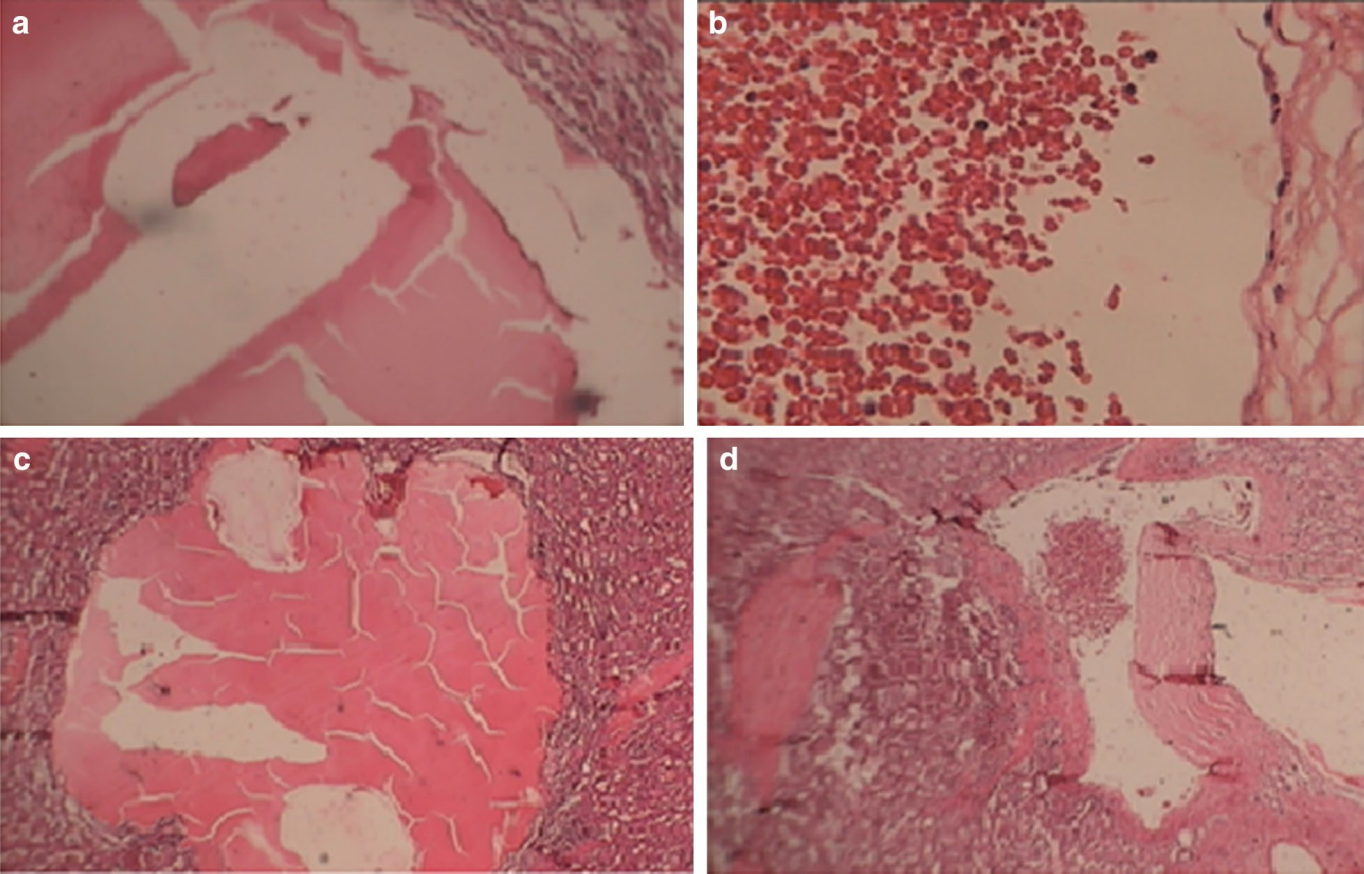
Histopathology of the spleen showed a large cavernous hemangioma occupying almost the entire spleen with large areas of infarction necrosis. **a** Blood-filled cystic spaces (cavernous hemangioma). **b** Cystic spaces lined by endothelial-like cells. **c, d** Dilated capillaries in the spleen containing red blood cells with endothelial lining

The resected specimen and imprint cytology was sent for pathology evaluation but it did not show any evidence of malignancy. Postoperative recovery of the patient was uneventful. Histopathology of the spleen showed a large cavernous hemangioma occupying almost the entire spleen with large areas of infarction necrosis (Fig. 3). Because of low back pain, he was referred to neurosurgeon and after assessments we found that the cause of his lumbar pain was hemangiomas of his vertebra. The last visit was 3 months ago and he was well without any complaint.

## Discussion

In this study we reported a rare case of a giant cavernous hemangioma with multiple hemangiomas in vertebra. To our knowledge this is the first report of concurrent of cavernous hemangioma of the spleen and hemangioma of the vertebra. The most common benign primary neoplasm in spleen is hemangioma [1, 2]. Cavernous hemangioma of the spleen is considered as a rare disorder with less than 100 reports so far [4] splenic hemangioma can be manifested as single or be a part of generalized angiomatosis including Klippel–Trenaunay–Weber syndrome, Von Hippel-Lindau disease and Sturge-Weber syndrome [13, 14]. In our study the CECT of the abdomen showed a large, lobulated mass with multiple hypodense round lesions in different sizes in its counter in anatomic site of spleen, suggestive of splenic hemangioma or metastasis. Hemangiomas are rarely identified with imaging examinations [1]. In line of our study, George Chatzoulis et al. in 2008 reported the co-existence of a giant splenic hemangioma and multiple hepatic hemangiomas in a 42-year-old female. They indicated the controversial therapeutic dilemma between resection and embolization of giant hemangiomas in their case report [15]. In other study by Elzbieta Gawrych et al. in 2012 reported a concurrent of splenic hemangioma and vascular malformation of the lower extremity in a child [16]. In consistent to our study they performed splenectomy as a surgical management for the patient. Hemangioma of the vertebra is a benign disease which does not require treatment in case the patient has no clear symptoms. It has been shown that more than 50 % of VH cases are presented with painful symptoms [12]. In patients with obvious symptoms treatment and surgical operation are recommended [8, 17]. However, there has been controversial view about surgical excision related to operation leads to bleeding or large trauma [18, 19]. In addition, MRI is sufficient to identify the location of the hemangioma. Moreover it can also show the spinal cord compression degree, so this is considered as a fundamental tool in surgical plan and prognosis. In this regard, In 2009 Toldo et al. reported a family with familial cerebral cavernous malformations coexistence with cutaneous, retinal, and hepatic lesions. In their study they indicated the significant role of spinal MRI in the diagnosis of spinal and vertebral cavernous angiomas. In their study they revealed that spinal MRI detected five cases with spinal cavernous angiomas either alone or associated with vertebral hemangiomas [20].

## Conclusions

In this study we reported a rare case that was suspicious to malignancy. After evaluation we found that the patient has a giant, cavernous hemangioma of the spleen presenting as a massive splenomegaly and multiple hemangiomas in his vertebrae. At the end this is important to consider splenic hemangioma in differential diagnosis of splenomegaly and the concurrent of the splenic hemangioma with vertebral hemangioma indicates the need for urgent therapeutic procedures as the condition is so critical.

## Consent

Written informed consent was obtained from the patient for publication of this case report and any accompanying images.
